# Tumor BRCA1, RRM1 and RRM2 mRNA Expression Levels and Clinical Response to First-Line Gemcitabine plus Docetaxel in Non-Small-Cell Lung Cancer Patients

**DOI:** 10.1371/journal.pone.0003695

**Published:** 2008-11-11

**Authors:** Ioannis Boukovinas, Chara Papadaki, Pedro Mendez, Miquel Taron, Dimitris Mavroudis, Anastasios Koutsopoulos, Maria Sanchez-Ronco, Jose Javier Sanchez, Maria Trypaki, Eustathios Staphopoulos, Vassilis Georgoulias, Rafael Rosell, John Souglakos

**Affiliations:** 1 “Theagenion” Cancer Hospital of Thessaloniki, Thessaloniki, Greece; 2 Laboratory of Tumor Cell Biology, School of Medicine, University of Crete, Heraklion, Greece; 3 Catalan Institute of Oncology, Hospital Germans Trias i Pujol, Ctra Canyet, Badalona, Barcelona, Spain; 4 Pangaea Biotech, USP Dexeus University, C/Sabino Arana 5, Barcelona, Spain; 5 Department of Medical Oncology, University General Hospital of Heraklion, Crete, Greece; 6 Department of Pathology, University General Hospital of Heraklion, Crete, Greece; 7 University of Alcala de Henares, Alcala de Henares, Spain; 8 Autonomous University of Madrid, Madrid, Spain; Karolinska Institutet, Sweden

## Abstract

**Background:**

Overexpression of RRM1 and RRM2 has been associated with gemcitabine resistance. BRCA1 overexpression increases sensitivity to paclitaxel and docetaxel. We have retrospectively examined the effect of RRM1, RRM2 and BRCA1 expression on outcome to gemcitabine plus docetaxel in advanced non-small-cell lung cancer (NSCLC) patients.

**Methodology and Principal Findings:**

Tumor samples were collected from 102 chemotherapy-naïve advanced NSCLC patients treated with gemcitabine plus docetaxel as part of a randomized trial. RRM1, RRM2 and BRCA1 mRNA levels were assessed by quantitative PCR and correlated with response, time to progression and survival. As BRCA1 levels increased, the probability of response increased (Odds Ratio [OR], 1.09: *p* = 0.01) and the risk of progression decreased (hazard ratio [HR], 0.99; *p* = 0.36). As RRM1 and RRM2 levels increased, the probability of response decreased (RRM1: OR, 0.97; *p* = 0.82; RRM2: OR, 0.94; *p*<0.0001) and the risk of progression increased (RRM1: HR, 1.02; *p* = 0.001; RRM2: HR, 1.005; *p* = 0.01). An interaction observed between BRCA1 and RRM1 allowed patients to be classified in three risk groups according to combinations of gene expression levels, with times to progression of 10.13, 4.17 and 2.30 months (*p* = 0.001). Low BRCA1 expression was the only factor significantly associated with longer time to progression in 31 patients receiving cisplatin-based second-line therapy.

**Conclusions:**

The mRNA expression of BRCA1, RRM1 and RRM2 is potentially a useful tool for selecting NSCLC patients for individualized chemotherapy and warrants further investigation in prospective studies.

## Introduction

Non-small-cell lung cancer (NSCLC) remains the leading cause of cancer death[1], with little improvement in survival regardless of the type of chemotherapy used, either in combination or as single agents[2]. Combinations of third-generation cytotoxic agents, such as taxanes, vinorelbine and gemcitabine, with cisplatin have emerged as new standards. In several phase III clinical trials in advanced NSCLC, the combination of platinum with taxanes attained median survival times of 8–11 months and 1-year survival of 31–46%[3]. Non-platinum-based combinations with gemcitabine plus docetaxel or paclitaxel have yielded a similar survival benefit with a more favorable toxicity profile[4], [5]. In order to further improve survival, a phase III trial of customized cisplatin according to ERCC1 mRNA levels in stage IV NSCLC was carried out. Patients in the control arm received cisplatin plus docetaxel, while in the genotypic arm, patients with low ERCC1 levels received cisplatin plus docetaxel and those with high levels received gemcitabine plus docetaxel[6]. Although objective response was higher in the genotypic arm than in the control arm (50.7% vs 39.3%), this did not translate to improved survival. The British Thoracic Oncology Group trial (BTOG1) also found no association between ERCC1 levels and survival in advanced NSCLC patients treated with docetaxel plus carboplatin[7]. Retrospective studies of stage IV NSCLC have reported that patients with low ERCC1 or RRM1 mRNA levels had a median survival up to 15 months when treated with gemcitabine plus cisplatin, with more significant differences in survival according to RRM1 levels[8], [9], [10]. A feasibility study of customized treatment in NSCLC patients with high ERCC1 and low ribonucleotide reductase subunit M1 (RRM1) mRNA expression found that gemcitabine plus docetaxel could be the optimal combination for this subgroup of patients[11].

RRM1 and RRM2 are encoded by different genes on separate chromosomes and their mRNAs are differentially expressed throughout the cell cycle. Reduced expression of let-7 microRNA (miRNA) is frequently observed in NSCLC[12] and often leads to overexpression of RRM1 and RRM2[13]. Resistance to gemcitabine has been associated with both RRM1 and RRM2 overexpression[14], [15]. Small interfering RNA targeting RRM2 enhanced chemosensitivity to gemcitabine in pancreatic adenocarcinoma[16]. In our study of metastatic lung adenocarcinoma patients treated with gemcitabine plus docetaxel, patients with low levels of both RRM1 and RRM2 had a significantly higher response rate (60% vs 14.2%), time to progression (9.9 vs 2.3 months), and overall survival (15.4 vs 3.6 months) than patients with high levels of both genes[17].

A close correlation has also been observed between expression levels of RRM1 and BRCA1[18], [19], [20], and the loss of let-7 has been shown to upregulate *BRCA1* as well as *RRM1* and *RRM2* [13]. In addition, both *BRCA1* and *RRM1* are upregulated in the SV40 T/t-antigen signature[21]. BRCA1 expression confers differential chemosensitivity in cancer cell lines[22], [23]. Ovarian cancer patients in the lowest terciles of BRCA1 expression showed sensitivity to cisplatin and resistance to paclitaxel and docetaxel, while those in the highest terciles had resistance to cisplatin and sensitivity to paclitaxel and docetaxel[23]. Low levels of BRCA1 also correlated with increased survival in NSCLC patients treated with gemcitabine plus cisplatin[18].

In order to validate our previous findings on RRM1 and RRM2[17] and to further investigate the role of BRCA1 in taxane sensitivity and resistance, we retrospectively analyzed a series of tumor samples from advanced NSCLC patients treated with gemcitabine plus docetaxel in a randomized phase III trial carried out by the Hellenic Oncology Research Group (HORG)[5].

## Methods

### Patients

Tumor samples were collected from primary tumors from patients with histologically confirmed inoperable stage IIIB and IV NSCLC, who were included in the experimental arm of a HORG randomized trial carried out from April 1999 to September 2002 [5] (no trial registration required before 2005). Eligibility criteria have been previously reported. The study was approved by the Ethics Committees of the participating hospitals, and all patients gave their signed informed consent prior to study entry. Patients received first-line gemcitabine (Gemzar®; Eli Lilly, Indianapolis, IN, USA) 1000 mg/m^2^ on days 1 and 8 and docetaxel (Taxotere®; Sanofi-Aventis, Collegeville, NJ, USA) 100 mg/m^2^ on day 8, with human granulocyte colony-stimulating factor support every 3 weeks, as previously described. Patient evaluation was performed at baseline and after every three cycles of chemotherapy[5].

### Study design

The present study was a retrospective analysis of the prognostic value of BRCA1, RRM1 and RRM2 mRNA expression in NSCLC patients treated with first-line gemcitabine plus docetaxel. All available tumor biopsies of the primary tumor with more than 100 cells per section were included in the analysis. All efficacy results were assessed for all enrolled patients on an intent-to-treat basis.

### Gene expression analysis

All paraffin-embedded tumors were reviewed by two independent pathologists to define the most appropriate tumor area for microdissection to ensure a minimum of 90% of tumor cells. Malignant cells were procured using an Eppendorf piezoelectric microdissector (Eppendorf, Hamburg, Germany). RNA was purified by trizol LS method (Invitrogen, Carlsbad, CA, USA) followed by isopropanol precipitation and DNase treatment (Ambion, Austin, TX, USA). cDNA synthesis was performed using SuperScript III reverse transcriptase (Invitrogen, Carlsbad, CA, USA). Relative quantification of gene expression was performed using the ABI Prism 7900HT Sequence Detection System (Applied Biosystems, Foster City, CA, USA). (For further details on the gene expression analysis, see Text S1).

### Statistical analyses

Besides analyzing the expression levels of each gene as a continuous variable, gene expression was also categorized in terciles in order to explore the risk trend of the gene variables and in order to easily identify groups of gene expression levels with different risk. Responses were recorded according to the RECIST criteria[24]. Median time to tumor progression and overall survival were calculated from the start of treatment to the first documented disease progression or death, respectively.

The potential association between baseline characteristics, response and gene expression levels were compared with either the two-sided Fisher's exact test or the Chi-square test for categorical variables and the Kruskal-Wallis test for continuous variables. The normality of continuous variables was verified with a Kolmogorov-Smirnov test. The Spearman test was used to evaluate the correlation between BRCA1, RRM1 and RRM2 mRNA expression. All potential risk factors for response were evaluated in a univariate analysis, and a multivariate logistic regression analysis, with adjusted odd ratios and their 95% confidence intervals (CI), was used to evaluate which of the factors had a significant influence on response. The Hosmer-Lemeshow likelihood test was used to assess the goodness of fit.

The association of risk factors with time-to-event endpoints was analyzed with the log-rank test, and the Kaplan-Meier method was used to plot the corresponding time-to-progression and survival curves. A univariate Cox regression analysis, with hazard ratios and 95% CIs, was used to assess the association between each potential prognostic factor and survival and time to progression. These factors were then included in a multivariate Cox proportional hazards regression model with a stepwise procedure (both forward and backward) to evaluate the independent significance of different variables on survival and time to progression. The likelihood ratio test was used to assess the goodness of fit, and the Wald's test was used to assess the coefficient significance. In the case of potential multiple comparisons, the *p*-values were corrected with the Bonferroni correction.

All statistical calculations were performed with SPSS, version 15.0 (SPSS, Inc., Chicago, IL, USA). Two-sided *p*-values of less than 0.05 were considered significant.

## Results

### Patient characteristics and clinical outcome

**Figure 1:**
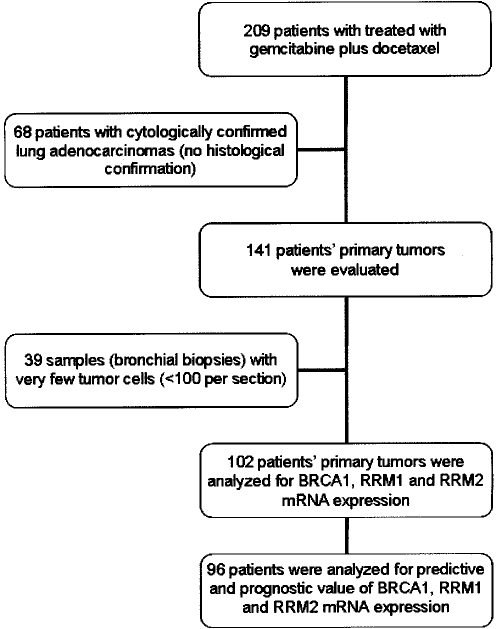
Chart showing the process of obtaining archival paraffin-embedded tumor biopsies for the assessment of BRCA1, RRM1 and RRM2 mRNA expression. Two hundred and nine patients were treated with gemcitabine plus docetaxel as part of a phase III randomized trial in advanced NSCLC[5]. Tumor biopsy was obtained from a total of 102 patients; 68 patients were ruled out because only cytological specimens were available, and 39 bronchial biopsies contained too few tumor cells for analysis. mRNA expression analysis was feasible in tumor samples from 96 of 102 patients.

In the original randomized trial[5], 209 NSCLC patients were treated with gemcitabine plus docetaxel; 107 were not included in the present study due to lack of tumor tissue (Fig. 1). Clinical data and samples from primary tumors were available for 102 patients, who were included in the present study. Amplification of BRCA1, RRM1 and RRM2 was successful in 96 samples. Eighty-one were adenocarcinomas, ten squamous cell carcinomas, and five large-cell carcinomas. Patient characteristics are shown in Table 1. In the original trial, the response rate was 30%, time to progression 4 months, and median survival 9 months[5]. Outcome for the 96 patients assessed in the present study was similar: response rate 30.5%, time to progression 4.2 months, and median survival 10.5 months.

**Table 1:** Patient characteristics.

		N (%)	Median (range)
**Total patients**		96 (100)	
**Gender**			
	**Male**	87 (90)	
	**Female**	9 (10)	
**ECOG Performance Status**			
	**0**	62 (65)	
	**1**	28 (29)	
	**2**	6 (6)	
**Stage**			
	**IIIB**	25 (26)	
	**IV**	71 (74)	
**Age (years)**		96	60 (37–76)
**BRCA1 median value**		96	3.64 (0–34.37)
**BRCA1 by terciles (T)**			
	**BRCA1 T1**	32	1.09 (0–2.31)
	**BRCA1 T2**	32	3.64 (2.33–7.81)
	**BRCA1 T3**	32	11.30 (8.07–34.37)
**RRM1 median value**		96	0.82 (0–325.23)
**RRM1 by terciles (T)**			
	**RRM1 T1**	32	0.26 (0–0.44)
	**RRM1 T2**	32	0.82 (0.45–1.10)
	**RRM1 T3**	32	2.18 (1.14–325.23)
**RRM2 median value**		96	27.16 (0.97–256.84)
**RRM2 by terciles (T)**			
	**RRM2 T1**	32	4.97 (0.97–13.93)
	**RRM2 T2**	32	27.16 (15.04–45.71)
	**RRM2 T3**	32	89.96 (46.08–265.84)

### BRCA1, RRM1 and RRM2 mRNA expression levels

Median mRNA expression levels were 3.64 (range 0–34.37) for BRCA1, 0.82 (range 0–325.23) for RRM1 and 27.16 (range 0.97–256.84) for RRM2 (Table 1). Expression levels did not follow a normal distribution (Fig. S1). There was no correlation between age, gender, PS, or disease stage and BRCA1, RRM1 or RRM2 mRNA levels. Significant correlations were observed overall between BRCA1 and RRM1 (ρ = 0.27; *p* = 0.008) and a non-significant trend to correlation between RRM1 and RRM2 (ρ = 0.19; *p* = 0.06) mRNA levels. There was also a significant inverse correlation between BRCA1 and RRM2 mRNA levels (ρ = −0.25; *p* = 0.02). Table 1 also shows the mRNA expression levels of the three genes according to terciles.

### Gene expression and response to treatment

In order to predict response to treatment, a logistic regression model was fitted for the expression of each gene as a continuous variable. As BRCA1 levels increased, the probability of response increased significantly (Odds Ratio [OR] = 1.09; 95% CI, 1.02–1.16; *p* = 0.01). In contrast, as RRM2 levels increased, the probability of response decreased significantly (OR = 0.94; 95% CI, 0.91–0.97; *p*<0.0001). A similar but non-significant trend was observed for RRM1 levels (OR = 0.97; 95% CI, 0.77–1.23; *p* = 0.82).

When responders were classified according to their gene expression levels by terciles, the majority of responders had high BRCA1 expression and low RRM2 expression: 58.6% in the highest tercile of BRCA1 expression (*p* = 0.002) and 72.4% in the lowest tercile of RRM2 expression (*p*<0.0001) (Table 2).

**Table 2:** Univariate and multivariate analyses for response to treatment.

		CR+PR N (%)	SD+PD N (%)	*p*	Univariate OR (95% CI)	*p*	Multivariate OR (95% CI)	*p*
**BRCA1**				0.002				
	**T1**	8 (27.6)	23 (34.8)		0.31 (0.11–0.89)	0.03	0.54 (0.13–2.22)	0.40
	**T2**	4 (13.8)	28 (42.4)		0.13 (0.04–0.44)	0.001	0.22 (0.05–1.01)	0.05
	**T3**	17 (58.6)	15 (22.7)		1		1	
**RRM1**				0.56				
	**T1**	12 (41.4)	20 (30.3)		1		1	
	**T2**	9 (31)	23 (34.8)		0.65 (0.23–1.87)	0.43	1.43 (0.34–6.03)	0.62
	**T3**	8 (27.6)	23 (34.8)		0.58 (0.20–1.70)	0.32	0.95 (0.22–4.13)	0.94
**RRM2**				<0.001				
	**T1**	21 (72.4)	10 (15.2)		1		1	
	**T2**	7 (24.1)	25 (37.9)		0.13 (0.04–0.41)	<0.001	0.20 (0.06–0.73)	0.02
	**T3**	1 (3.4)	31 (47)		0.02 (0.002–0.13)	<0.001	0.02 (0.002–0.17)	<0.001
**ECOG PS**				0.02				
	**0**	24 (82.8)	38 (57.6)		1		1	
	**1–2**	5 (17.2)	28 (42.4)		0.28 (0.10–0.83)	0.02	0.44 (0.11–1.69)	0.23
**STAGE**				0.31				
	**IIIB**	10 (34.5)	15 (22.7)		1		1	
	**IV**	19 (65.5)	51 (77.3)		0.56 (0.21–1.46)	0.23	0.45 (0.12–1.74)	0.25

CR, complete response; PR, partial response; SD, stable disease; PD, progressive disease; OR, odds ratio; T, terciles; PS, performance status.

The univariate logistic regression analysis revealed that low RRM2 expression, ECOG PS 0, and high BRCA1 expression were significantly associated with a higher probability of response (Table 2). In the multivariate logistic regression analysis of these variables together with RRM1 and disease stage, only low RRM2 expression emerged as an independent predictive factor for response (Table 2).

### Gene expression and time to progression

The univariate analysis for time to progression revealed that the only clinical variable associated with time to progression was PS (Hazard Ratio [HR] for PS 1–2, 1.55; 95% CI, 0.99–2.41; *p* = 0.05) (Table 3). The univariate analysis for time to progression according to gene expression levels as continuous variables showed that as RRM1 and RRM2 values increased, the risk of progression increased significantly: RRM1 (HR, 1.02; 95% CI, 1.01–1.02; *p* = 0.001); RRM2 (HR, 1.005; 95% CI, 1.001–1.008; *p* = 0.01). However, as BRCA1 levels increased, the risk of progression decreased (HR, 0.99; 95% CI, 0.95–1.02; *p* = 0.36).

**Table 3:** Median time to progression according to gene expression, ECOG PS and disease stage.

		TTP mos (95% CI)	Log-rank *p*	Univariate HR (95% CI)	Cox *p*	Multivariate HR (95% CI)	Cox *p*
**BRCA1**			0.25				
	**T1**	3 (1.9–4.1)		1.51 (0.91–2.49)	0.11	1.51 (0.86–2.65)	0.15
	**T2**	3.6 (2.2–4.9)		1.33 (0.80–2.22)	0.28	1.10 (0.62–1.95)	0.74
	**T3**	5.5 (3.1–7.9)		1		1	
**RRM1**			0.27				
	**T1**	6 (0.2–11.8)		1		1	
	**T2**	3.7 (0.6–6.6)		1.20 (0.72–1.97)	0.49	1.39 (0.82–2.37)	0.22
	**T3**	3.3 (2.2–4.4)		1.51 (0.91–2.51)	0.11	1.72 (0.99–3)	0.06
**RRM2**			0.03				
	**T1**	8.7 (4.9–12.4)		1		1	
	**T2**	3.6 (2–5.2)		1.28 (0.77–2.13)	0.35	1.02 (0.57–1.80)	0.95
	**T3**	2.7 (1.6–3.8)		1.93 (1.16–3.22)	0.01	1.55 (0.89–2.71)	0.12
**PS**			0.05				
	**0**	5.23 (3.99–6.48)		1		1	
	**1–2**	2.70 (1.99–3.41)		1.55 (0.99–2.41)	0.05	1.62 (1.01–2.59)	0.05
**STAGE**			0.13				
	**IIIB**	5.50 (1.86–9.15)		1		1	
	**IV**	4.17 (3.05–5.28)		1.45 (0.89–2.36)	0.13	1.43 (0.86–2.38)	0.17

TTP, time to progression; HR, hazard ratio; T, tercile; PS, performance status.

When gene expression levels were categorized by terciles, the risk of progression was greater for patients in the intermediate and highest terciles of RRM1 and RRM2 than for those in the lowest tercile: RRM1 intermediate tercile (HR, 1.20; 95% CI, 0.72–1.97; *p* = 0.49); RRM1 highest tercile (HR, 1.51; 95% CI, 0.91–2.51; *p* = 0.11); RRM2 intermediate tercile (HR, 1.28; 95% CI, 0.77–2.13; *p* = 0.35); RRM2 highest tercile (HR, 1.93; 95% CI, 1.16–3.22; *p* = 0.01) (Table 3). The risk of progression was greater for patients in the intermediate and lowest tercile of BRCA1 than for those in the highest tercile: BRCA1 intermediate tercile (HR, 1.33; 95% CI, 0.80–2.22; *p* = 0.28); BRCA1 lowest tercile (HR, 1.51; 95% CI, 0.91–2.49; *p* = 0.11) (Table 3). Time to progression according to gene expression by terciles is shown in Table 3.

A multivariate model was fitted with the variables examined in the univariate setting.

When interaction terms were examined to check whether they significantly improved the fit, none was significant except for BRCA*RRM1, which gave a significance of *p* = 0.02 to the model without the interaction term (Table S1). The multivariate model was then stratified by RRM1 (Table S2) and without disease stage. In this model, patients in the lowest tercile of RRM2 continued to have the lowest risk of progression, independently of RRM1 levels.

Patients were classified in three groups according to risk of progression, based on the interaction observed between RRM1 and BRCA1. Twenty-four patients were in the low-risk group (intermediate BRCA1+low RRM1; high BRCA1+low RRM1; high BRCA1+intermediate RRM1); 42 patients were in the intermediate-risk group (low BRCA1+low RRM1; intermediate BRCA1+high RRM1; high BRCA1+high RRM1); and 30 patients were in the high-risk group (low BRCA1+intermediate RRM1; intermediate BRCA1+intermediate RRM1; low BRCA1+high RRM1).

**Figure 2:**
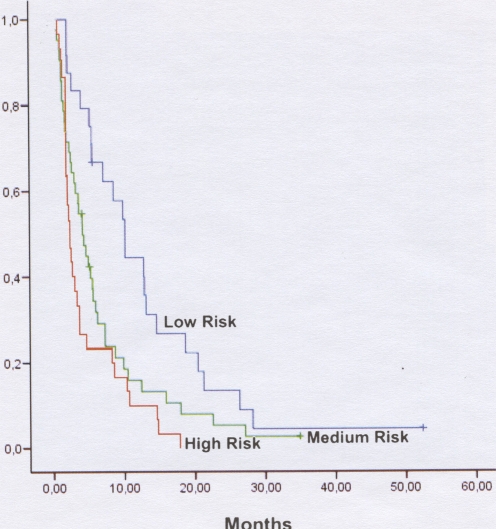
Time to progression according to risk groups. Patients were classified in three groups according to risk of progression, based on the interaction observed between RRM1 and BRCA1. Twenty-four patients were in the low-risk group (intermediate BRCA1+low RRM1; high BRCA1+low RRM1; high BRCA1+intermediate RRM1); 42 patients were in the intermediate-risk group (low BRCA1+low RRM1; intermediate BRCA1+high RRM1; high BRCA1+high RRM1); and 30 patients were in the high-risk group (low BRCA1+intermediate RRM1; intermediate BRCA1+intermediate RRM1; low BRCA1+high RRM1). The median time to progression was 10.13 months (95% CI, 7.65–12.62) for patients in the low-risk group, 4.17 months (95% CI, 72.90–5.44) for patients in the intermediate-risk group, and 2.30 months (95% CI, 1.76–2.84) for patients in the high-risk group (*p* = 0.001) (See also Tables S2, S3).

The median time to progression was 10.13 months (95% CI, 7.65–12.62) for patients in the low-risk group, 4.17 months (95% CI, 72.90–5.44) for patients in the intermediate-risk group, and 2.30 months (95% CI, 1.76–2.84) for patients in the high-risk group (*p* = 0.001) (Tables S2, S3; Fig. 2).

### Gene expression and survival

In the univariate analysis of survival, the only significant clinical variable was PS (HR for PS 1–2, 1.94; 95% CI, 1.21–3.12; *p* = 0.005) (Table 4). As RRM1 and RRM2 values increased, the risk of death increased: RRM1 (HR, 1.01; 95% CI, 1.00–1.02; *p* = 0.005); RRM2 (HR, 1.004; 95% CI, 1.00–1.008; *p* = 0.06). However, as BRCA1 levels increased, the risk of death decreased (HR, 0.99; 95% CI, 0.96–1.03; *p* = 0.60). When gene expression levels were categorized in terciles, the same pattern of increased risk of death was observed for higher levels of both RRM1 and RRM2 and lower levels of BRCA1 (Table 4). In the multivariate model including all the variables from the univariate analysis, only PS emerged as a significant factor for survival (Table 4).

**Table 4:** Median survival according to gene expression, PS and disease stage.

		MS mos (95% CI)	Log-rank *p*	Univariate HR (95% CI)	Cox *p*	Multivariate HR (95% CI)	Cox *p*
**BRCA1**			0.37				
	**T1**	12 (6.4–17.6)		1.28 (0.74–2.21)	0.37	1.39 (0.75–2.56)	0.29
	**T2**	8.5 (2.2–14.8)		1.51 (0.85–2.88)	0.16	1.48 (0.79–2.79)	0.22
	**T3**	12.7 (5.7–19.7)		1		1	
**RRM1**			0.39				
	**T1**	12 (5.7–18.3)		1		1	
	**T2**	10.6 (5.3–15.8)		1.17 (0.68–2.02)	0.57	1.37 (0.76–2.46)	0.29
	**T3**	11.2 (2.9–19.5)		1.47 (0.85–2.56)	0.17	1.73 (0.94–3.18)	0.08
**RRM2**			0.48				
	**T1**	15.2 (10.1–20.2)		1		1	
	**T2**	9.3 (3.7–14.8)		1.15 (0.66–2.01)	0.62	0.74 (0.39–1.40)	0.35
	**T3**	6.6 (1.5–11.7)		1.40 (0.81–2.42)	0.23	0.91 (0.49–1.71)	0.77
**PS**			0.005				
	**0**	15.17 (10.15–20.18)		1		1	
	**1–2**	6.30 (4.16–8.44)		1.94 (1.21–3.12)	0.006	2.07 (1.25–3.43)	0.005
**STAGE**			0.10				
	**IIIB**	17.93 (2.25–33.62)		1		1	
	**IV**	10.27 (5.85–14.68)		1.57 (0.92–2.63)	0.10	1.45 (0.85–2.47)	0.18

MS, median survival; HR, hazard ratio; T, tercile; PS, performance status.

### Gene expression and second-line treatment

**Figure 3:**
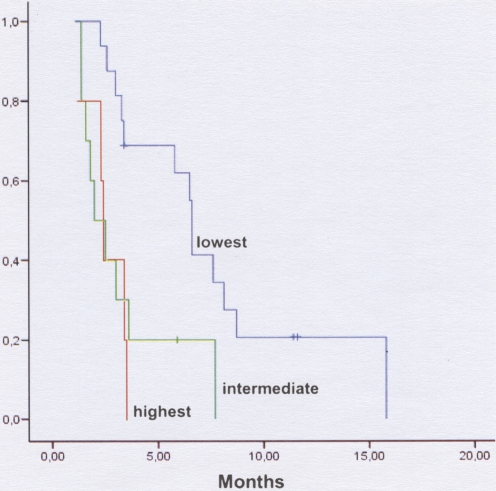
Time to progression after first-line treatment according to BRCA1 terciles. In contrast to the pattern observed with first-line therapy, low levels of BRCA1 were significantly associated with the lowest risk of progression to second-line therapy. Median time to progression was 6.60 months for patients in the lowest tercile, 2 months for those in the intermediate tercile, and 2.40 months for those in the highest tercile of BRCA1 expression (*p* = 0.004) (Table 5). (For further details, see text.)

Second-line therapy was administered in 31 patients, 90.3% of whom received cisplatin-based chemotherapy. There were no differences in gene expression levels between patients receiving and those not receiving second-line therapy. Time to progression for all 31 patients calculated from the start of second-line therapy was 3.40 months (95% CI, 2.73–4.07). In contrast to the pattern observed with first-line therapy, low levels of BRCA1 were significantly associated with the lowest risk of progression to second-line therapy. Median time to progression was 6.60 months for patients in the lowest tercile, 2 months for those in the intermediate tercile, and 2.40 months for those in the highest tercile of BRCA1 expression (*p* = 0.004) (Table 5, Fig. 3). BRCA1 mRNA expression emerged as the only significant factor in both the univariate and multivariate analyses of time to progression in the 31 patients receiving second-line therapy (Table 6).

**Table 5:** Time to progression after first-line treatment according to gene expression levels in 31 patients receiving second-line therapy.

		N	TTP mos (95% CI)	*p*
**BRCA1**				0.004
	**T1**	16	6.60 (6.42–6.78)	
	**T2**	10	2.00 (0.92–3.09)	
	**T3**	5	2.40 (2.19–2.62)	
**RRM1**				0.49
	**T1**	10	2.60 (1.67–3.53)	
	**T2**	13	5.80 (1.64–9.96)	
	**T3**	8	3.50 (2.67–4.33)	
**RRM2**				0.76
	**T1**	5	6.50 (0.11–12.90)	
	**T2**	9	5.80 (0–12.23)	
	**T3**	17	3 (1.92–4.08)	

TTP, time to progression; T, tercile.

**Table 6:** Univariate and multivariate analyses of time to progression after first-line therapy for 31 patients receiving second-line treatment.

		N	Univariate HR (95% CI)	Cox *p*	Multivariate HR (95% CI)	Cox *p*
**BRCA1**						
	**T1**	16	1		1	
	**T2**	10	3.35 (1.33–8.44)	0.01	4.37 (1.53–12.49)	0.006
	**T3**	5	4.78 (1.50–15.26)	0.008	6.61 (1.86–23.50)	0.004
**RRM1**						
	**T1**	10	1		1	
	**T2**	13	0.76 (0.32–1.85)	0.55	0.47 (0.14–1.60)	0.23
	**T3**	8	0.54 (0.19–1.53)	0.24	0.37 (0.10–1.35)	0.13
**RRM2**						
	**T1**	5	1		1	
	**T2**	9	0.72 (0.21–2.49)	0.61	1.22 (0.24–6.14)	0.81
	**T3**	17	1.01 (0.32–3.10)	0.99	1.82 (0.42–8.01)	0.43
**PS**						
	**0**	22	1		1	
	**1–2**	9	1.61 (0.67–3.83)	0.28	1.78 (0.64–4.91)	0.27

HR, hazard ratio; PS, performance status.

## Discussion

The present study has found an inverse correlation between RRM2 mRNA expression and response to gemcitabine plus docetaxel in advanced NSCLC patients. Patients with low RRM2 mRNA expression attained a significantly higher response rate and time to progression than those with high RRM2 expression. In addition, RRM2 mRNA expression was revealed as an independent predictive factor for response. These results confirm our earlier findings in a small cohort of lung adenocarcinomas treated with the same regimen[17]. Although median RRM2 levels were different in the two studies, possibly due to slight differences in patient populations or in some steps of the RNA extraction and PCR quantification procedures, the correlation between mRNA levels and clinical results was similar in both studies. Intriguingly, transgenic mice developed lung adenocarcinoma but not other tumors in the presence of RRM2 overexpression[25]. In earlier retrospective studies[8], [9], we found that high levels of RRM1 predicted longer survival in stage IV NSCLC patients treated with gemcitabine plus cisplatin but not in those treated with cisplatin-based regimens without gemcitabine.

The significant correlation between the top tercile of BRCA1 mRNA expression and improved response observed in the present study adds to the growing body of evidence that BRCA1 is a crucial mediator of DNA damage response[26]. Low BRCA1 expression confers increased sensitivity to cisplatin[22], [23], [27], [28] and etoposide[22] and resistance to antimicrotubule drugs, such as paclitaxel[22], [23], [28], docetaxel[23] and vinorelbine[22], while high BRCA1 expression leads to resistance to cisplatin[22], [23], [27], [28] and etoposide[22] and sensitivity to paclitaxel[22], [23], [28], docetaxel[23] and vinorelbine[22]. In the present study, patients with low BRCA1 mRNA expression had poor response and time to progression to first-line gemcitabine plus docetaxel; in contrast, they obtained the maximum benefit from second-line cisplatin-based treatment, attaining a median time to progression of 6.6 months.

Several layers of evidence show that the abrogation of BRCA1 function leads to resistance to antimicrotubule drugs. Spindle checkpoint defects are associated with resistance to taxanes and vinca alkaloids. Suppression of *Mad2* or *BubR1* in paclitaxel-treated breast cancer MCF-7 cells abolishes spindle checkpoint function, resulting in enhanced paclitaxel resistance[29]. In addition, downregulation of BRCA1 expression mediates paclitaxel resistance through premature inactivation of spindle checkpoint in MCF-7 cells via downregulation of BubR1[30].

*BRCA1* dysfunction is closely related to spindle checkpoint defects but not to G2 phase alterations. Indeed, a set of gene expression alterations due to the knockdown of endogenous BRCA1 has been identified in prostate (DU-145) and breast (MCF-7) cancer cells by DNA microarray analysis[31]. Various categories of genes are downregulated in BRCA1-knockdown cells, including genes involved in transcription and cell cycle regulation and in DNA replication and repair. BRCA1-short interference RNAs (siRNAs) also caused the downregulation of DNA topoisomerase II alpha (TOP2A), an enzyme involved in DNA replication and in both the DNA damage-responsive G2 checkpoint and the G2 decatenation checkpoint. This checkpoint requires TOP2A, ATR, WRN (Werner's syndrome helicase), and BRCA1; it is defective in cells with mutant *BRCA1*. However, in contrast to *BRCA1*-mutant cell lines, knockdown of wild-type BRCA1 did not attenuate this checkpoint but showed attenuation of the mitotic spindle checkpoint[31]. BRCA1 positively regulates the expression of many genes involved in the spindle checkpoint, such as *Bub1* and *BubR1*. Consistent with these findings, cells pretreated with BRCA1-siRNAs failed to arrest in metaphase after treatment with nocodazole[31].

Interestingly, BRCA1-siRNAs also caused downregulation of metabolism genes, including *RRM2* and *dihydrofolate reductase (DHFR)* [31]. Moreover, an integrated gene signature from multiple transgenic models of epithelial cancers intrinsic to the functions of the Simian virus 40T/t-antigens is composed of genes regulating cell replication, proliferation and DNA repair. BRCA1 is overexpressed in three T/t-antigenic transgenic mouse models (breast, lung, prostate), as are other genes, including *Bub1b*, *TOP2A*, *DHFR*, *thymidylate synthase (TS)*, and *RRM1* [21].

The limited efficacy of current chemotherapy approaches is epitomized in metastatic NSCLC, where time to progression ranges from 5.2 to 5.5 months, with different combinations of chemotherapy, such as docetaxel plus cisplatin[32] or new compounds like pemetrexed (an inhibitor of TS and DHFR) plus cisplatin[33]. Therefore, in spite of the retrospective nature of the present study, a time to progression of 10 months in 24 of 96 patients in the low-risk group (high BRCA1 and low RRM1 levels), treated with docetaxel plus gemcitabine, represents a promising new landmark that merits validation in a prospective trial of customized chemotherapy. These clinical findings are similar to those obtained with EGFR tyrosine kinase inhibitors in NSCLC patients harboring *EGFR* mutations. In the only prospective study reported of treatment with gefitinib in 31 NSCLC patients with *EGFR* mutations, a time to progression of 9.2 months was attained[34].

From the present study, we cannot determine whether the mRNA expression of BRCA1, RRM1 or RRM2 could have a prognostic – as well as a predictive – role. In early-stage, chemonaive, resected NSCLC patients, BRCA1 mRNA expression was the only independent prognostic variable[20]. Similarly, high mRNA expression of the BRCA1-interacting protein BACH1/Brip1 has been found in aggressive breast cancers[35]. In addition, BRCC36 has been shown to be present in the BRCA1-RAP80 complex [36]and is overexpressed in breast cancer, where it confers radioresistance[37]. This highlights the possibility that BRCA1 – or several interacting partners – can confer poor prognosis as well as resistance to cisplatin or other DNA-damaging agents.

In summary, our findings indicate that the efficacy of gemcitabine plus docetaxel can be improved when customized according to the mRNA expression of BRCA1, RRM1 and RRM2. It is intriguing to speculate that BRCA1 could become an important predictive marker for customizing pemetrexed plus cisplatin in patients with low BRCA1 levels, since BRCA1 could be a surrogate of DHFR and TS levels[21], [31]. Prospective studies of customized chemotherapy based on the expression of these genes have been opened.

## Supporting Information

Figure S1 Box plots showing mRNA expression values for BRCA1, RRM1 and RRM2. Numerical values shown on each box plot are values that differ from the median. These numerical values have the probability of belonging to the distribution of these genes.
(2.25 MB TIF)

Table S1 Interactions for time to progression
(0.05 MB DOC)

Table S2 Multivariate analysis of time to progression stratified by RRM1
(0.04 MB DOC)

Table S3 Median time to progression stratified by RRM1
(0.04 MB DOC)

Text S1 Boukovinas et al - BRCA1
(0.04 MB DOC)
