# Supplementary figures and images for: Tumor BRCA1, RRM1 and RRM2 mRNA Expression Levels and Clinical Response to First-Line Gemcitabine plus Docetaxel in Non-Small-Cell Lung Cancer Patients
Source: PLoS One. 2008 Nov 11;3(11):e3695. doi: 10.1371/journal.pone.0003695 (PMC2579656; doi:10.1371/journal.pone.0003695)

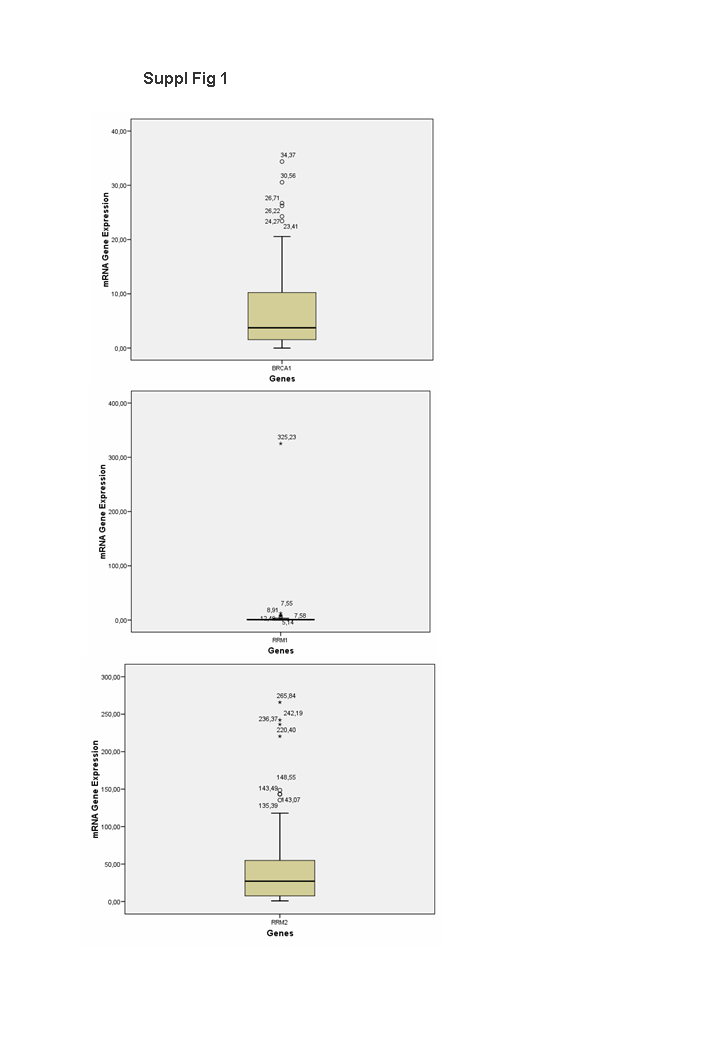

Supplement: Figure S1 — Box plots showing mRNA expression values for BRCA1, RRM1 and RRM2. Numerical values shown on each box plot are values that differ from the median. These numerical values have the probability of belonging to the distribution of these genes. (2.25 MB TIF) [file pone.0003695.s001.tif]
