# Supplementary material for: Tumor BRCA1, RRM1 and RRM2 mRNA Expression Levels and Clinical Response to First-Line Gemcitabine plus Docetaxel in Non-Small-Cell Lung Cancer Patients
Source: PLoS One. 2008 Nov 11;3(11):e3695. doi: 10.1371/journal.pone.0003695 (PMC2579656; doi:10.1371/journal.pone.0003695)
Supplement: Table S1 — Interactions for time to progression (0.05 MB DOC) [file pone.0003695.s002.doc]

**Table S1.** Interactions for time to progression

|  |  | **Multivariate Analysis**  **HR (95% CI)** | **Cox *p*** |
| --- | --- | --- | --- |
| **BRCA1** |  |  |  |
|  | **T1** | 0.82 (0.34-2.01) | 0.67 |
|  | **T2** | 0.57 (0.19-1.73) | 0.32 |
|  | **T3** | 1 |  |
| **RRM1** |  |  |  |
|  | **T1** | 1 |  |
|  | **T2** | 0.37 (0.12-1.14) | 0.08 |
|  | **T3** | 1.62 (0.68-3.84) | 0.28 |
| **RRM2** |  |  |  |
|  | **T1** | 1 |  |
|  | **T2** | 1.40 (0.75-2.61) | 0.30 |
|  | **T3** | 1.82 (1.01-3.27) | 0.05 |
| **PS** |  |  |  |
|  | **0** | 1 |  |
|  | **1-2** | 1.70 (1.05-2.75) | 0.03 |
| **STAGE** |  |  |  |
|  | **IIIB** | 1 |  |
|  | **IV** | 1.38 (0.82-2.32) | 0.22 |
| **BRCA1*RRM1** |  |  |  |
|  | **BRCA1T3*RRM1T1** | 1 |  |
|  | **BRCA1T1*RRM1T2** | 5.46 (1.30-22.94) | 0.02 |
|  | **BRCA1T2*RRM1T2** | 7.06 (1.52-32.65) | 0.01 |
|  | **BRCA1T1*RRM1T3** | 1.54 (0.41-5.82) | 0.53 |
|  | **BRCA1T2*RRM1T3** | 0.78 (0.20-2.99) | 0.71 |
