# Supplementary material for: Tumor BRCA1, RRM1 and RRM2 mRNA Expression Levels and Clinical Response to First-Line Gemcitabine plus Docetaxel in Non-Small-Cell Lung Cancer Patients
Source: PLoS One. 2008 Nov 11;3(11):e3695. doi: 10.1371/journal.pone.0003695 (PMC2579656; doi:10.1371/journal.pone.0003695)
Supplement: Table S2 — Multivariate analysis of time to progression stratified by RRM1 (0.04 MB DOC) [file pone.0003695.s003.doc]

**Table S2.** Multivariate analysis of time to progression stratified by RRM1

|  |  | **RRM1 T1** | | **RRM1 T2** | | **RRM1 T3** | |
| --- | --- | --- | --- | --- | --- | --- | --- |
|  |  | **HR (95% CI)** | **Cox *p*** | **HR (95% CI)** | **Cox *p*** | **HR (95% CI)** | **Cox *p*** |
| **BRCA1** |  |  |  |  |  |  |  |
|  | **T1** | 0.72 (0.23-2.23) | 0.57 | 4.59 (1.35-15.58) | 0.01 | 1.80 (0.63-5.10) | 0.27 |
|  | **T2** | 0.35 (0.09-1.38) | 0.13 | 4.72 (1.44-15.44) | 0.01 | 0.64 (0.24-1.74) | 0.38 |
|  | **T3** | 1 |  | 1 |  | 1 |  |
| **RRM2** |  |  |  |  |  |  |  |
|  | **T1** | 1 |  | 1 |  | 1 |  |
|  | **T2** | 2.44 (0.81-7.35) | 0.11 | 2.30 (0.74-7.15) | 0.15 | 1.15 (0.34-3.83) | 0.82 |
|  | **T3** | 1.21 (0.35-4.22) | 0.77 | 3.07 (0.98-9.59) | 0.05 | 1.55 (0.60-3.96) | 0.37 |
| **PS** |  |  |  |  |  |  |  |
|  | **0** | 1 |  | 1 |  | 1 |  |
|  | **1-2** | 2.27 (0.98-5.26) | 0.06 | 0.77 (0.31-1.92) | 0.58 | 2.98 (1.21-7.35) | 0.02 |
