# Supplementary material for: Tumor BRCA1, RRM1 and RRM2 mRNA Expression Levels and Clinical Response to First-Line Gemcitabine plus Docetaxel in Non-Small-Cell Lung Cancer Patients
Source: PLoS One. 2008 Nov 11;3(11):e3695. doi: 10.1371/journal.pone.0003695 (PMC2579656; doi:10.1371/journal.pone.0003695)
Supplement: Table S3 — Median time to progression stratified by RRM1 (0.04 MB DOC) [file pone.0003695.s004.doc]

**Table S3.** Median time to progression stratified by RRM1

| **RRM1 T1** | | | | | **RRM1 T2** | | | **RRM1 T3** | | |
| --- | --- | --- | --- | --- | --- | --- | --- | --- | --- | --- |
|  |  | **N (%)** | **TTP**  **mos (95%CI)** | ***p*** | **N (%)** | **TTP**  **mos (95%CI)** | ***p*** | **N (%)** | **TTP**  **mos (95%CI)** | ***p*** |
| **BRCA1** |  |  |  | 0.75 |  |  | 0.007 |  |  | 0.68 |
|  | **T1** | 16 (50) | 4.17 (3.71-4.62) |  | 10 (31.3) | 2 (1.28-2.72) |  | 6 (18.8) | 2.40 (1.08-3.72) |  |
|  | **T2** | 6 (18.8) | 8.50 (0-17.42) |  | 14 (43.8) | 2 (1.10-3.30) |  | 12 (37.5) | 3.60 (1.13-6.07) |  |
|  | **T3** | 10 (31.3) | 9.87 (2.69-17.05) |  | 8 (25) | 12.80 (0-26.82) |  | 14 (43.8) | 3.57 (0-7.85) |  |
| **RRM2** |  |  |  | 0.49 |  |  | 0.06 |  |  | 0.76 |
|  | **T1** | 14 (43.8) | 9.87 (7.24-12.50) |  | 7 (21.9) | 14.63 (6.23-23.04) |  | 11 (34.4) | 4.97 (0.83-9.10) |  |
|  | **T2** | 11 (34.4) | 4.33 (0.34-8.33) |  | 13 (40.6) | 3.63 (1.52-5.75) |  | 8 (25) | 3 (2.51-3.49) |  |
|  | **T3** | 7 (21.9) | 3.20 (1.92-4.48) |  | 12 (37.5) | 2.20 (1.52-2.88) |  | 13 (40.6) | 3 (1.30-5.30) |  |
