# Supplementary material for: Tumor BRCA1, RRM1 and RRM2 mRNA Expression Levels and Clinical Response to First-Line Gemcitabine plus Docetaxel in Non-Small-Cell Lung Cancer Patients
Source: PLoS One. 2008 Nov 11;3(11):e3695. doi: 10.1371/journal.pone.0003695 (PMC2579656; doi:10.1371/journal.pone.0003695)
Supplement: Text S1 — Boukovinas et al - BRCA1 (0.04 MB DOC) [file pone.0003695.s005.doc]

**Text S1**

***Gene expression analysis***

The pellet of microdissected cells was resuspended in 200μl RNA lyses buffer[1] and incubated at 60°C for 16 hours until the tissue was completely solubilized. RNA was purified by trizol LS (Invitrogen, Carlsbad, CA, USA) extractions followed by isopropanol precipitation and DNase (DNase Free, Ambion, Austin, TX, USA) treatment in order to avoid genomic DNA contamination of the samples. cDNA synthesis was performed in a final volume of 22 μl using SuperScript III reverse transcriptase according to the manufacturer’s protocol (Invitrogen, Carlsbad, CA, USA). The QPCR reaction was performed using 2.5 μl of template cDNA to 6.25 μl Taqman Universal Master Mix (Applied Biosystems, Foster City, CA, USA) with the addition of specific primers and probe for each gene and adjusted with DEPC water to a final volume of 12.5μl per reaction. Quantification of gene expression was performed using the ABI Prism 7900HT Sequence Detection System (Applied Biosystems, Foster City, CA, USA). All primers and probe sets were designed to spread into an exon-exon junction, as has been previously reported[2,3].

Relative gene expression quantification was performed according to the comparative Ct method using β-actin as an endogenous control and commercial RNA controls (Stratagene, La Jolla, CA, USA) as calibrators. In all experiments, only triplicates with a standard deviation of the Ct value <0.25 were accepted. In addition, genomic DNA contamination was excluded by non-reverse transcript RNA for each sample analyzed.

**References**

1. Specht K, Richter T, Muller U, Walch A, Werner M, et al. (2001) Quantitative gene expression analysis in microdissected archival formalin-fixed and paraffin-embedded tumor tissue. Am J Pathol 158: 419-429.

2. Souglakos J, Boukovinas I, Taron M, Mendez P, Mavroudis D, et al. (2008) Ribonucleotide reductase subunits M1 and M2 mRNA expression levels and clinical outcome of lung adenocarcinoma patients treated with docetaxel/gemcitabine. Br J Cancer 98: 1710-1715.

3. Taron M, Rosell R, Felip E, Mendez P, Souglakos J, et al. (2004) BRCA1 mRNA expression levels as an indicator of chemoresistance in lung cancer. Hum Mol Genet 13: 2443-2449.

**Figure Legends**

**Figure S1.** Box plots showing mRNA expression values for BRCA1, RRM1 and RRM2. Numerical values shown on each box plot are values that differ from the median. These numerical values have the probability of belonging to the distribution of these genes.

**Table Legends**

**Table S1.** Interactions for time to progression

**Table S2.** Multivariate analysis of time to progression stratified by RRM1

**Table S3.** Median time to progression stratified by RRM1
